# Discriminative analysis of schizophrenia using support vector machine and recursive feature elimination on structural MRI images: Erratum

**DOI:** 10.1097/01.md.0000504794.22466.69

**Published:** 2016-10-21

In the article “Discriminative analysis of schizophrenia using support vector machine and recursive feature elimination on structural MRI images”,^[1]^ which appeared in Volume 95, Issue 30 of *Medicine*, some funding information was originally omitted. The article has since been corrected online.
